# From screening to treatment: the new landscape of diabetic kidney disease

**DOI:** 10.1186/s12916-022-02537-4

**Published:** 2022-10-07

**Authors:** C. Elena Cervantes, Mohamad Hanouneh, Bernard G. Jaar

**Affiliations:** 1 Department of Medicine, Division of Nephrology, Johns Hopkins University School of Medicine, 1830 E. Monument Street, Baltimore, MD 21205 USA; 2 Nephrology Center of Maryland, Baltimore, USA; 3 Department of Epidemiology, The Johns Hopkins Bloomberg School of Public Health, Baltimore, USA; 4 The Welch Center for Prevention, Epidemiology, and Clinical Research, Baltimore, USA

**Keywords:** Chronic kidney disease, Diabetes mellitus, Diabetic kidney disease, Screening, Treatment, Stroke

## Abstract

Globally, diabetes mellitus is the leading cause of chronic kidney disease (CKD), and it is predicted to increase in the following years. Despite its high prevalence, CKD remains under diagnosed. In this *BMC Medicine* collection of articles on diabetic kidney disease (DKD), we place in context the importance of screening and early detection of DKD and the most accurate tools to monitor for optimal glycemic control in this his risk population. Further, we address this population's risk for severe complications such as stroke and all-cause mortality. We close this editorial by summarizing recent advances in management of this vulnerable population of patients with DKD, including guideline-directed medical therapy, novel treatments, and predictors of treatment failure.

## Background

The prevalence of diabetes worldwide in 2021 was a staggering 537 million people. This number is predicted to increase to 580 and 700 million cases by 2030 and 2045, respectively [1–3]. Further, the number of Americans with diabetes (37 million) was estimated at 10.5% of the population in 2018, which is also predicted to increase to 14% by 2030 [4]. According to the Centers for Disease Control and Prevention (CDC), 1 in 7 Americans had chronic kidney disease (CKD) in 2021, with diabetes being the leading cause in more than half of the cases [5]. The growing numbers of patients with CKD in the USA can be traced in large part to the burgeoning epidemic of diabetes. In fact, the USA is estimated to have more than 1 million patients with end-stage kidney disease by 2030, an increase of nearly 40% since 2015 [6]. Worldwide, in 2010, there were nearly 500 million adults with CKD with more than 75% of them residing in low- and middle-income countries (LMICs) [7]. As a result of these trends, society as a whole but specifically healthcare systems and families will face an increasing financial burden.

### From screening to treatment advances

Between 2011 and 2014, only about 21% of people with CKD stages 3 and 4 with diabetes were aware of their diagnosis [5]. This issue led the National Kidney Foundation to label CKD as “the under-recognized public health crisis” in the USA [8]. However, one of the challenges of diagnosing CKD is that patients often remain asymptomatic until the disease has reached an advanced stage [9, 10]. In this collection, George C. and colleagues highlight the disproportionately high burden of CKD and diabetes in LMICs [11]. Some factors that play a role include lack of disease awareness, late referrals, and the cost of screening, among others. The authors found that screening for CKD in people with diabetes is generally infrequent in LMICs, which is contributed by the lack of a fully subsidized healthcare program for people with non-dialysis CKD, resulting in high out-of-pocket costs. In order to reduce the burden and progression of diabetes, Kidney Disease Improving Global Outcomes (KDIGO) guidelines recommend that all patients with diabetes undergo annual serum creatinine-based estimated glomerular filtration rate (eGFR) and urine tests to evaluate for albuminuria [12]. However, in LMICs, the dipstick test for urinary protein remains the most frequently used due to its cost-effectiveness and accessibility. As the authors point out, the inclusion of serum cystatin C in the eGFR estimation equations has proven to be more accurate than creatinine-based estimations. However, it remains to be explored whether this is true across different populations in the globe. Finally, prediction models to estimate the risk of undiagnosed CKD and the risk of progression of CKD in diabetics are being increasingly used in the USA. Even though they allow identifying those most likely to benefit from more aggressive interventions, they are population specific. Notably, albuminuria or impaired eGFR alone are associated with 10-year mortality of 18% and 24%, respectively, in people with diabetes. However, mortality is more than additive when both abnormalities are present, reaching nearly 50% [13, 14].

Glycemic monitoring and intensive glycemic control still play an important role in preventing diabetic kidney disease (DKD) in the early stages, although overall CKD progression and cardiovascular mortality may not be significantly reduced after the onset of DKD [15]. KDIGO guidelines recommend a target hemoglobin A1c (HbA1c) ranging from < 6.5 to < 8.0% unless the patient is at increased risk of hypoglycemia [12]. However, as Hassanein and Shafi pointed out in this collection, HbA1c is a less reliable marker for diabetes control due to the concomitant presence of anemia in people with DKD, particularly in those with advanced CKD [16]. Nevertheless, to date, it remains the gold standard method to monitor blood glucose control in this high-risk population. The authors present data showing that continuous glucose monitoring correlates with hemoglobin A1c and other indirect markers such as fructosamine, glycated albumin, and 1,5-anhydroglucitol in people with diabetes. But they also carry their own caveats. Proteinuria and hypoalbuminemia, common findings in CKD patients, can affect fructosamine and glycated albumin levels. Regarding 1,5-anhydroglucitol, it is highly dependent on tubular glucose reabsorption, which could be variable in patients with CKD. As one can realize, none of these markers are reliable in a population with DKD. Therefore, we suggest continuous glucose monitoring as an emerging tool to achieve glycemic control, but certainly, more studies are required to validate its efficacy in this population [16].

Despite the alarming statistics about the projected rates of increase in diabetes and the room for improvement in screening for DKD, among US adults with diabetes, all-cause mortality has declined by 20% [5]. This has been attributed to intensified, targeted therapies and lower cardiovascular disease (CVD). However, people with diabetes still have higher mortality than the rest of the population, suggesting the role of other risk factors. Previous literature has shown that estimated glucose disposal rates (eGDRs), a surrogate of insulin resistance, predict all-cause mortality in type 2 diabetes, independent of DKD. Therefore, in this collection, Penno and colleagues observed that the more insulin resistance, the higher the CVD risk. This relationship was independent of traditional CVD risk factors. Furthermore, eGDRs significantly affected mortality in people with non-DKD and normoalbuminuric DKD but not in those with albuminuric DKD. The authors suggest that mortality could be mediated by albuminuria in people with albuminuric DKD and not by insulin resistance [17].

Overall, patients with CKD are over 5 times more likely to die from cardiovascular causes than to progress to ESKD [9]. Cardiovascular morbidity also plays an essential role in people with diabetes. A major public health concern is the impact of coronary artery disease and stroke on disability and healthcare costs. In this collection, Kaze and colleagues looked at factors that increase the risk of stroke in the Action to Control Cardiovascular Risk in Diabetes (ACCORD) study. The authors reported that the higher the albuminuria (above 30 mg/g) and the lower the eGFR (below 60 mL/min/1.73 m^2^), the higher was the risk of stroke. Further, compared to no CKD, worsening CKD stages defined by the KDIGO criteria were associated with an increased risk of stroke [18].

As outlined by Hanouneh and colleagues in this collection, treatment can be targeted to the stage of DKD. Therefore, in the early stages, intensive glycemic control is critical, with metformin and sodium-glucose cotransporter 2 inhibitors (SGLT2is) being excellent candidates when the eGFR is above 30 ml/min per 1.73 m^2^ [19]. Glucagon-like peptide-1 receptor agonists are an additional tool to manage hyperglycemia in this high-risk population. Further, optimal blood pressure control should be achieved with a blood pressure goal below 130/80 mmHg, ideally with a renin–angiotensin–aldosterone system (RAAS) blockade agent [12, 20]. In addition, KDIGO guidelines suggest that individuals with DKD follow a diet low in sodium (< 2 g/day), maintain a protein intake of 0.8 g/kg/day for those not on dialysis, and exercise for at least 150 min per week as tolerated [12]. Fortunately, new treatment strategies have emerged to help curve the epidemic of DKD. SGLT2is were found to have significant cardiovascular benefits and to be renoprotective, independent of the blood-glucose-lowering effect [21–23]. Additionally, finerenone, a highly selective nonsteroidal mineralocorticoid receptor antagonist (MRA), produces potent anti-inflammatory and antifibrotic effects beyond RAAS blockade agents and significantly reduces proteinuria and DKD progression [24]. These therapeutic advances are summarized in Table 1.

**Table 1:** Recent chronic kidney disease randomized clinical trials of SGLT2is and selective nonsteroidal mineralocorticoid receptor antagonist

Trial name	Intervention	Population size	Target population	Primary endpoint	Secondary endpoint	Median follow-up	Results
EMPA-REG [22]	Empagliflozin 10 mg daily or 25 mg daily vs placebo	7020	Type II diabetic patients with established CV disease and eGFR > 30 mL/min/1.73 m^2^ with body mass index < 45 and HgA1c 7–9%	Composite of death from cardiovascular causes, nonfatal myocardial infarction, or nonfatal stroke	Renal outcomes: Progression to macroalbuminuria, doubling serum creatinine, renal replacement therapy or death from renal disease	3.1 years	- Primary endpoint: 14% lower in canagliflozin group (hazard ratio, 0.86; 95.02% CI, 0.74 to 0.99) - Secondary endpoint: 39% lower in empagliflozin group (hazard ratio, 0.61; 95% CI, 0.53 to 0.70)
CREDENCE [23]	All patients on RAAS blockade agent Canagliflozin 100 mg daily s placebo	4401	Type II diabetic patients with eGFR 30–89 mL/min/1.73 m^2^ and urine albumin/creatinine ratio between > 300 and 5000 mg/g	Composite of end-stage kidney disease, doubling of serum creatinine, or kidney/cardiovascular-related death	Composite of cardiovascular death, myocardial infarction, stroke, and hospitalization for heart failure	2.62 years	- Primary endpoint: 30% lower in canagliflozin group (hazard ratio, 0.70; 95% CI, 0.59 to 0.82) - Secondary endpoint: 39% lower in canagliflozin group (hazard ratio, 0.61; 95% CI, 0.47 to 0.80)
DAPA-CKD [21]	All patients on RAAS blockade agent for at least four weeks Dapagliflozin 10 mg daily vs placebo	4304	eGFR between 25 and 75 mL/min/1.73 m^2^ and urinary albumin-to-creatinine ratio of 200 to 5000 mg/g 67% of patients had diabetes mellitus type 2	Composite of sustained decline in eGFR of at least 50%, end-stage kidney disease, or death from kidney or cardiovascular causes	Composite of death from cardiovascular causes or hospitalization for heart failure	2.4 years	- Primary endpoint: 39% lower in the dapagliflozin group (hazard ratio, 0.61; 95% CI, 0.51 to 0.72) - Primary endpoint in DKD participants: 36% lower in dapagliflozin group (hazard ratio 0.64; 95% CI, 0.52 to 0.79) - Primary endpoint in CKD without diabetes: 50% lower in dapagliflozin group (hazard ratio 0.50 95% CI, 0.35 to 0.72) - Secondary endpoint: 29% lower in dapagliflozin group (hazard ratio 0.71 (95% CI, 0.55 to 0.92)
FIDELIO-DKD [24]	All patients on maximally tolerated RAAS blockade agent Finerenone 10 to 20 mg daily vs placebo	5734	DKD patients with either urinary albumin-to-creatinine ratio of 30 to less than 300 mg/g, eGFR between 25 and 59 mL/min/1.73 m^2^ and diabetic retinopathy or had a urinary albumin-to-creatinine ratio between 300 and 5000 mg/g with eGFR of 25 to 74 mL/min/1.73 m^2^	Composite of kidney failure, a sustained eGFR decrease of at least 40%, or death from renal causes	Cardiovascular death, nonfatal myocardial infarction, nonfatal stroke, or heart failure-related hospitalization	2.6 years	- Primary endpoint: 18% lower in the finerenone group (hazard ratio, 0.82; 95% CI, 0.73 to 0.93) - Secondary endpoint: 14% lower in the finerenone group (hazard ratio, 0.86; 95% CI, 0.75 to 0.99)

*SGLT2is* Sodium/glucose cotransporter-2 inhibitors, *eGFR* Estimated glomerular filtration rate, *DKD* Diabetic kidney disease, *CKD* Chronic kidney disease, *HbA1c* Hemoglobin A1c

Importantly, a recent study highlighted in this series on DKD identified predictors of cardio-kidney complications and treatment failure in this high-risk population treated with SGLT2 inhibitors. Using a retrospective study design and analyzing data from a large administrative health claims database of a national insurer, Kovesdy and colleagues identified the following risk factors, such high baseline CVD risk profile, atrial fibrillation, peripheral arterial disease, and heart failure [25].

## Conclusion

The diagnosis and treatment of DKD have recently seen significant progress in slowing down CKD progression, reducing cardiovascular events, and reducing mortality, but we still have a lot to learn; progress is still needed to further reduce the progression of DKD and hopefully one day, halt the disease process altogether. In the meantime, we need to intensify our DKD screening efforts, making sure that every patient with diabetes is appropriately tested for albuminuria and reduced GFR, particularly in LMICs. Optimal management of diabetes remains a cornerstone of the primary prevention of DKD, with intensive glycemic control starting early in the course of diabetes and implementing adequate blood pressure control and lifestyle modifications. There is also room for improvement in uptake of proven treatment with RAAS blockade agents, in addition to novel therapies such as SGLT2is and the highly selective nonsteroidal MRA finerenone [26]. All these interventions can only be successful if we have full patient engagement in the process.

## Data Availability

Not applicable.
